# The Effect of Metformin and Carbohydrate-Controlled Diet on DNA Methylation and Gene Expression in the Endometrium of Women with Polycystic Ovary Syndrome

**DOI:** 10.3390/ijms24076857

**Published:** 2023-04-06

**Authors:** Elizabeth García-Gómez, Yadira Inés Gómez-Viais, Martin Mizael Cruz-Aranda, Luis Daniel Martínez-Razo, Christian Reyes-Mayoral, Lizeth Ibarra-González, Araceli Montoya-Estrada, Mauricio Osorio-Caballero, Otilia Perichart-Perera, Ignacio Camacho-Arroyo, Marco Cerbón, Enrique Reyes-Muñoz, Edgar Ricardo Vázquez-Martínez

**Affiliations:** 1 Consejo Nacional de Ciencia y Tecnología (CONACYT)-Unidad de Investigación en Reproducción Humana, Instituto Nacional de Perinatología (INPer)-Facultad de Química, Universidad Nacional Autónoma de México (UNAM), Mexico City 11000, Mexico; 2 Unidad de Investigación en Reproducción Humana, Instituto Nacional de Perinatología (INPer)-Facultad de Química, Universidad Nacional Autónoma de México (UNAM), Mexico City 11000, Mexico; 3 Clínica Para el Estudio de la Reproducción Humana (CERH) Bajío, Irapuato 36650, Mexico; 4 Departamento de Nutrición y Bioprogramación, Instituto Nacional de Perinatología (INPer), Mexico City 11000, Mexico; 5 Coordinación de Endocrinología Ginecológica y Perinatal, Instituto Nacional de Perinatología (INPer), Mexico City 11000, Mexico; 6 Departamento de Salud Sexual y Reproductiva, Instituto Nacional de Perinatología (INPer), Mexico City 11000, Mexico

**Keywords:** polycystic ovary syndrome, endometrium, DNA methylation, gene expression, metformin, dietary intervention, insulin pathway, endometrial receptivity

## Abstract

Polycystic ovary syndrome (PCOS) is an endocrine disease associated with infertility and metabolic disorders in reproductive-aged women. In this study, we evaluated the expression of eight genes related to endometrial function and their DNA methylation levels in the endometrium of PCOS patients and women without the disease (control group). In addition, eight of the PCOS patients underwent intervention with metformin (1500 mg/day) and a carbohydrate-controlled diet (type and quantity) for three months. Clinical and metabolic parameters were determined, and RT-qPCR and MeDIP-qPCR were used to evaluate gene expression and DNA methylation levels, respectively. Decreased expression levels of *HOXA10*, *GAB1*, and *SLC2A4* genes and increased DNA methylation levels of the *HOXA10* promoter were found in the endometrium of PCOS patients compared to controls. After metformin and nutritional intervention, some metabolic and clinical variables improved in PCOS patients. This intervention was associated with increased expression of *HOXA10*, *ESR1, GAB1*, and *SLC2A4* genes and reduced DNA methylation levels of the *HOXA10* promoter in the endometrium of PCOS women. Our preliminary findings suggest that metformin and a carbohydrate-controlled diet improve endometrial function in PCOS patients, partly by modulating DNA methylation of the *HOXA10* gene promoter and the expression of genes implicated in endometrial receptivity and insulin signaling.

## 1. Introduction

Polycystic ovary syndrome (PCOS) is an endocrinopathy that affects up to 20% of reproductive-aged women worldwide. PCOS is characterized by hyperandrogenism, polycystic ovaries morphology features, ovulatory dysfunction, and metabolic disorders [1,2]. Clinical features include oligo or anovulation, menstrual disorders, hirsutism, acne, obesity, insulin resistance (IR), hyperinsulinemia, dyslipidemia, arterial hypertension, and chronic inflammation [3]. Notably, PCOS is a frequent cause of anovulatory infertility with high miscarriages and low pregnancy rates [4]. PCOS is diagnosed based on the Rotterdam Criteria, a convention of rules of the European Society for Human Reproduction and Embryology/American Society for Reproductive Medicine (ESHRE/ASRM) that establish that a patient must exhibit at least two of three symptoms between oligo/anovulation, hyperandrogenism, and the polycystic ovaries to confirm the disease [5].

The ovulatory dysfunction of PCOS is caused by a dysregulated hypothalamic–pituitary–ovarian (HPO) axis that results in an elevated luteinizing hormone/follicle-stimulating hormone (LH/FSH) ratio, which induces androgen synthesis and the arrest of ovarian follicle development [6,7]. The resulting hyperandrogenism is associated with IR, hyperinsulinemia, and high circulating levels of free fatty acids. IR results in impaired metabolic signaling and disruption of the HPO axis, favoring the androgen synthesis while inhibiting the synthesis of sex hormone-binding globulin (SHGB), which increases the level of free testosterone [1,8].

In addition to the ovaries, the endometrium is also affected by PCOS, contributing to infertility. Endometrial dysfunction is associated with defective decidualization, impaired progesterone signaling, continuous cell proliferation, and high levels of inflammatory markers [4,9]. These alterations are related to differential gene expression during the proliferative and secretory phases of the menstrual cycle [10,11,12,13]. A reduction in the gene expression of transcriptional regulators such as homeobox A10 (HOXA10) [14,15] (essential for endometrial development and function) [16], decidualization indicators such as insulin-like growth factor binding protein 1 (IGFBP-1) [9], and endometrial receptivity marker proteins such as leukemia inhibitory factor (LIF), progestagen-associated endometrial protein (PAEP), and glutathione peroxidase 3 (GPX3) (all considered essential for endometrial development and function) has been reported [11,17].

Genes involved in insulin signaling also exhibit a reduced expression, including those encoding glucose transporters GLUT1 and GLUT4, known as solute carrier family 2 member 1 (*SLC2A1*) and *SLC2A4*, respectively, insulin receptor substrate 1 (IRS1), and GRB2-associated-binding protein 1 (GAB1) [11,18,19]. GAB1, a docking protein closely related to the insulin receptor substrate, and GLUT4 are important proteins in insulin signaling and glucose metabolism in the human endometrium [18,20]. Furthermore, the altered expression of estrogen receptors ERα and ERβ (encoded by *ESR1* and *ESR2*, respectively), androgen receptor (AR) [21,22], and cellular proliferation regulator genes such as Paired box 6 (*PAX6*) has been documented [23].

Due to the proposed heterogeneous origin of PCOS and the influence of environmental factors and lifestyle on its development, epigenetic mechanisms have emerged as key players in the pathogenesis of the disease. In diverse tissues and cells of women with PCOS, such as peripheral blood, leukocytes, granulosa cells, the ovaries, hypothalamus, skeletal muscle, and fat tissue, altered patterns of transcription and DNA methylation correlated with clinical and biochemical characteristics of the disease [24,25,26,27]. DNA methylation changes have been observed in promoters of genes involved in insulin signaling, such as insulin receptor gene (*INSR*) in adipose tissue and peripheral blood [28], endometrial receptivity, such as *LIF* in ovary granulosa cells [29], and steroid metabolism, as is the case of *CYP19A1* (cytochrome P450 family 19 subfamilies A member 1) in ovarian tissue [30]. To our knowledge, a comprehensive analysis of DNA methylation levels in the endometrium of PCOS patients is lacking, and the existing information is scarce and inconclusive [31,32]. Only partial DNA methylation of the *INRS* gene promoter has been reported in endometrial samples of PCOS patients without finding a relationship with disease clinical manifestations [31].

In treating PCOS, lifestyle interventions (diet and physical activity), combined oral contraceptives, pharmacologic ovulation stimulation, and insulin-sensitizing drugs are commonly used. Among the last, metformin therapy in PCOS counteracts metabolic dysfunction and infertility by improving hormonal parameters and IR, restoring ovulation and periodicity of the menstrual cycles [2,18,33].

Some studies have reported increased mRNA levels of some genes involved in insulin signaling and hyperplasia in the endometrium of PCOS patients after metformin treatment and lifestyle changes [19,34,35,36], but its epigenetic effects are unidentified. Only in endometrial cancer is the precedent that metformin displays anti-tumoral properties, modifying DNA methylation and gene expression by modulating the activity of S-adenosylhomocysteine hydrolase [37,38]. However, the metformin effect on gene expression and epigenetic regulation in the endometrium of PCOS patients is widely unknown.

Particularly, the capacity of intervention with metformin and diet to modulate epigenetic and gene expression changes related to endometrial functionality in PCOS is still unexplored. Therefore, this study aimed to analyze the effect of metformin treatment and a carbohydrate-controlled diet (type and quantity) on DNA methylation and expression of selected genes related to endometrial function in endometrial tissue from patients with PCOS. We first evaluated the expression and DNA methylation levels of *HOXA10*, *PAX6*, *ESR1*, *ESR2*, *IGFBP1*, *GAB1*, *SLC2A4*, and *IRS1* genes, as well as global DNA methylation levels in the endometrium of PCOS patients and women without the disease (control group). Then, eight of the PCOS patients underwent intervention with metformin (1500 mg/day) and a carbohydrate-controlled diet (type and quantity) for three months. We analyzed clinical variables, gene expression, and DNA methylation levels before and after the intervention in these women. Our preliminary findings suggest that metformin and a carbohydrate-controlled diet improve endometrial function in PCOS patients, partly by modulating DNA methylation of the *HOXA10* gene promoter and the expression of genes implicated in endometrial receptivity and insulin signaling.

## 2. Results

### 2.1. Demographic and Clinical Characteristics of Participants

All the women with PCOS recruited in the present study had polycystic ovaries (as revealed on ultrasound), oligomenorrhea, and infertility. The analysis of several clinical and biochemical variables in the early proliferative phase of the menstrual cycle revealed that PCOS patients showed higher serum levels of androstenedione, estradiol, LH/FSH ratio, and testosterone, as well as free androgen index (FAI) and Homeostatic Model Assessment of Insulin Resistance (HOMA-IR) index than control women (Table 1).

### 2.2. HOXA10, GAB1, and SLC2A4 Genes Are Differentially Expressed in the Endometrium of PCOS Patients and Women without the Disease

**Figure 1:**
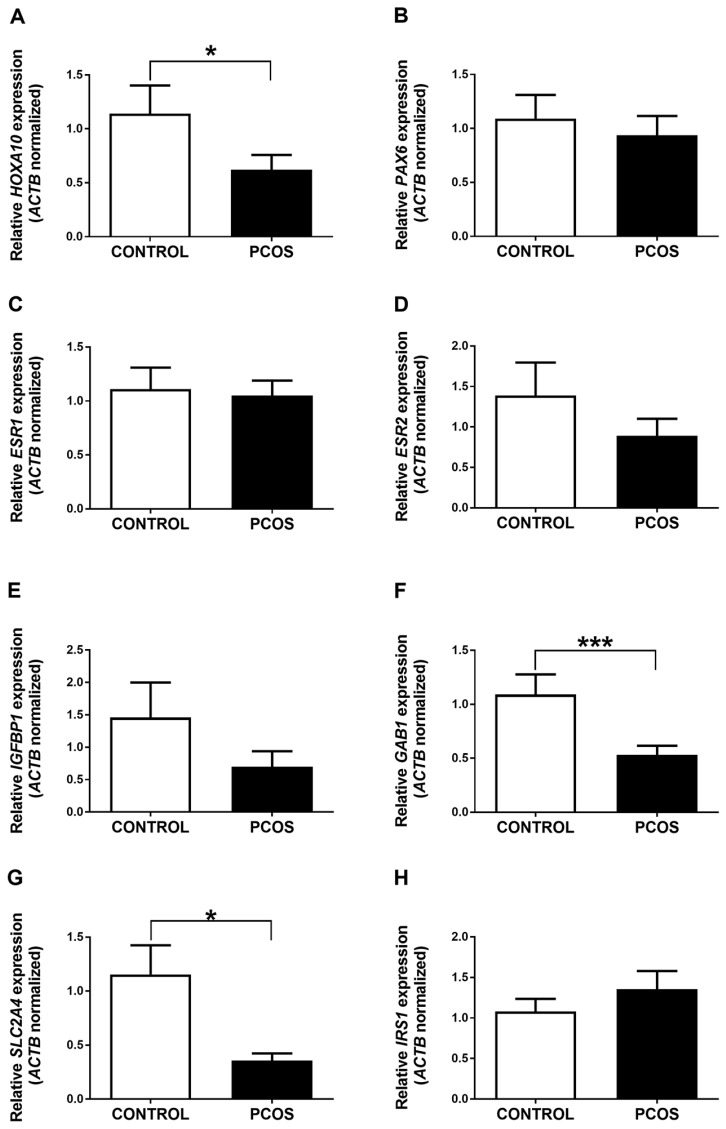
Expression of genes involved in endometrium function and insulin signaling in patients diagnosed with PCOS. Relative expression levels of *HOXA10* (**A**), *PAX6* (**B**), *ESR1* (**C**), *ESR2* (**D**), *IGFBP1* (**E**), *GAB1* (**F**), *SLC2A4* (**G**), and *ISR1* (**H**) are shown for healthy women (CONTROL) and PCOS patients. Data were obtained using the ΔΔCt method, normalizing mRNA levels with *ACTB*. Results are expressed as mean ± SE. * *p* ≤ 0.05, *** *p* ≤ 0.001.

The expression of genes associated with the endometrial function (*HOXA10*, *PAX6*, *ESR1*, *ESR2*, and *IGFBP1*) and insulin signaling (*GAB1*, *SLC2A4*, and *IRS1*) was evaluated in the mid-proliferative endometrium of PCOS patients and women without the disease (Figure 1). Results showed that gene expression of *HOXA10*, *GAB1*, and *SLC2A4* was significantly lower in the endometrium of PCOS women than in controls (Figure 1A,F,G). On the other hand, the gene expression of *PAX6*, *ESR1*, *ESR2*, *IGFBP1*, and *IRS1* was not statistically different between both study groups (Figure 1).

### 2.3. The Promoter of the HOXA10 Gene Is Differentially Methylated in the Endometrium of PCOS Patients and Women without the Disease

**Figure 2:**
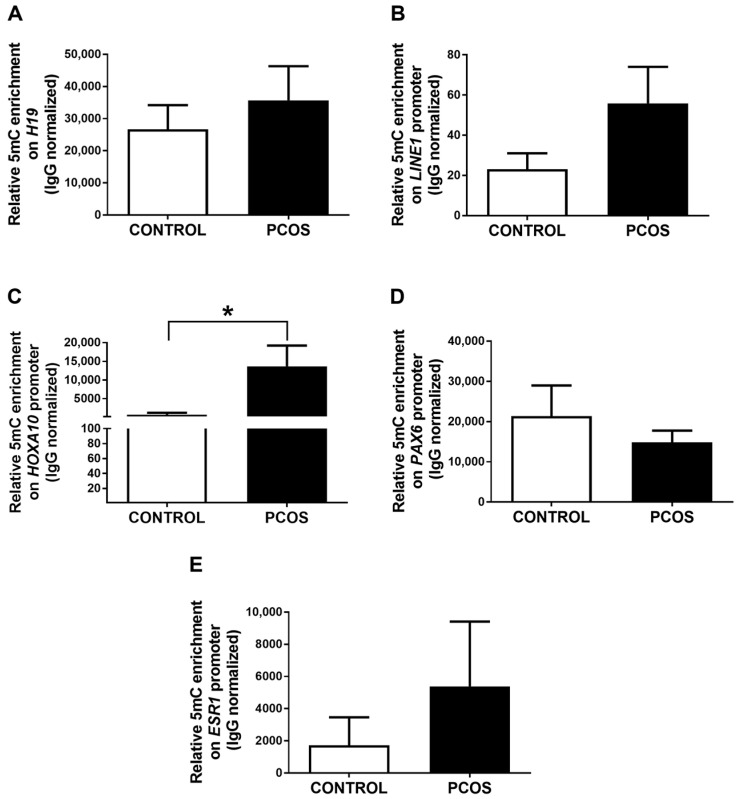
Content of DNA methylation at global markers and promoters of genes related to endometrium function in PCOS patients and controls. The relative levels of 5-methylcytosine (5mC) enrichment on *H19* gene (**A**), *LINE-1* (**B**), and promoters of genes *HOXA10* (**C**), *PAX6* (**D**), and *ESR1* (**E**) are shown for healthy women (CONTROL) and PCOS patients without treatment (PCOS). Data were obtained using MeDIP-qPCR and analyzed with the ΔΔCt method, normalizing with IgG enrichment. Results are expressed as mean ± SE. * *p* ≤ 0.05 vs. PCOS.

To determine the possible role of DNA methylation in the gene expression changes observed between the endometrium of PCOS patients and women without the disease, we evaluated markers of global DNA methylation (promoter of the imprinted gene *H19* and the repetitive region *LINE-1*) using methylated DNA immunoprecipitation (MeDIP). We found no differences in the levels of DNA methylation of *H19* and *LINE-1* between both study groups (Figure 2A,B). In addition, we analyzed the enrichment of this epigenetic mark on the promoters of the genes whose expression was previously determined. Interestingly, higher levels of DNA methylation at the *HOXA10* gene promoter were observed in the endometrium of women with PCOS compared to controls (Figure 2C). These findings are consistent with the lower expression of the *HOXA10* gene detected in the endometrium of PCOS patients compared to women without the disease (Figure 1A). We found no changes in the DNA methylation levels at the *PAX6* and *ESR1* gene promoters among both study groups (Figure 2D,E). We did not detect DNA methylation enrichment at the promoter of *ESR2*, *IGFBP1*, *GAB1*, *SLC2A4*, and *IRS1* genes in our experimental conditions in any of the study groups.

### 2.4. Intervention with Metformin and a Carbohydrate-Controlled Diet Improves Hormonal and Metabolic Profiles in PCOS Women

Eight of the PCOS patients recruited in the present study underwent intervention with metformin and a carbohydrate-controlled diet for three months (PCOS + MET group; Material and Methods). Five of eight patients documented at least one menstrual cycle during the intervention; however, we cannot discard the possible effect of medroxyprogesterone acetate (used to induce menstruation in PCOS patients) in these findings. Moreover, we did not find significant changes in the size and number of ovarian cysts after the intervention.

We observed a significant decrease in the BMI, androstenedione, estradiol, insulin, and HOMA-IR index in PCOS patients after the metabolic intervention (Table 2). These results suggest that metformin and carbohydrate restriction benefit the metabolic and hormonal profile of PCOS women.

### 2.5. Intervention with Metformin and a Carbohydrate-Controlled Diet Induces the Expression of HOXA10, ESR1, GAB1, and SLC2A4 Genes in the Endometrium of PCOS Women

**Figure 3:**
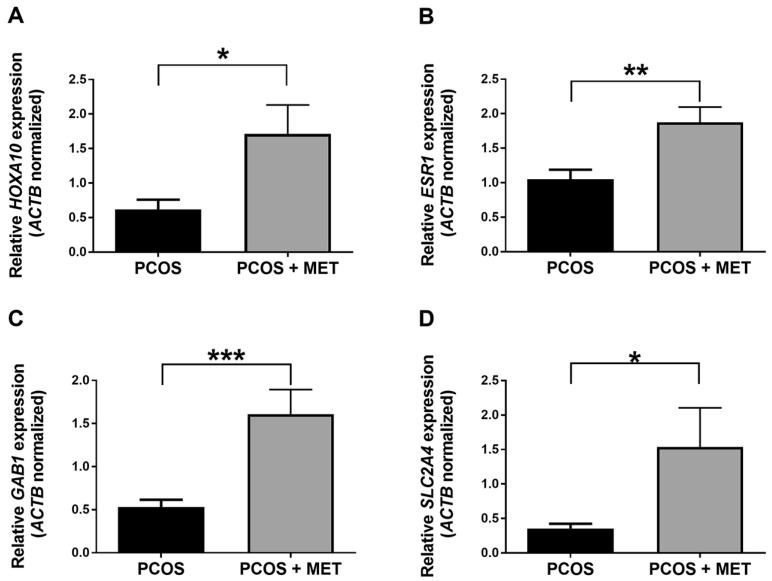
Effect of intervention with metformin and a carbohydrate-controlled diet on the expression of *HOXA10*, *ESR1*, *GAB1*, and *SLC2A4* genes in patients diagnosed with PCOS. Relative expression levels of *HOXA10* (**A**), *ESR1* (**B**), *GAB1* (**C**), and *SLC2A4* (**D**) are shown for PCOS patients without treatment (PCOS) and after intervention (PCOS + MET). Data were obtained using the ΔΔCt method, normalizing mRNA levels with *ACTB*. Results are expressed as mean ± SE. * *p* ≤ 0.05, ** *p* ≤ 0.01, *** *p* ≤ 0.001 vs. PCOS.

Results showed that the expression of *HOXA10*, *ESR1*, *GAB1*, and *SLC2A4* genes was significantly higher in the PCOS + MET group than in the PCOS group (Figure 3), suggesting that the pharmacologic and dietary intervention induced the expression of these genes in the endometrium of PCOS women. On the other hand, the gene expression of *PAX6*, *ESR2*, *IGFBP1*, and *IRS1* was not statistically different between both the study groups.

### 2.6. Effect of Intervention with Metformin and a Carbohydrate-Controlled Diet on DNA Methylation in the Endometrium of PCOS Women

**Figure 4:**
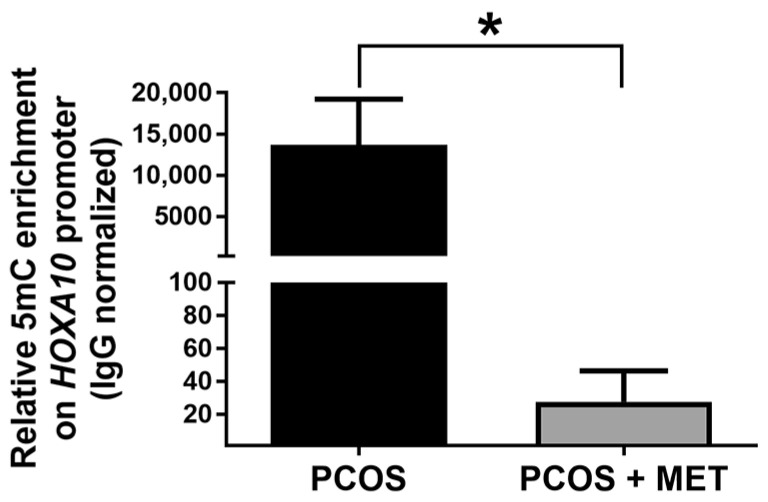
Effect of intervention with metformin and a carbohydrate-controlled diet on the content of DNA methylation at the promoter of *HOXA10* gene in PCOS patients. The relative level of 5-methylcytosine (5mC) enrichment on the promoter of *HOXA10* is shown for PCOS patients without treatment (PCOS) and after intervention (PCOS + MET). Data were obtained using MeDIP-qPCR and analyzed with the ΔΔCt method, normalizing with IgG enrichment. Results are expressed as mean ± SE. * *p* ≤ 0.05 vs. PCOS.

Endometrial samples obtained from women with PCOS subjected to metformin and dietary intervention showed no statistical differences in DNA methylation levels of *LINE-1* and the promoter of *H19*, *PAX6*, and *ESR1* genes compared to those obtained before the intervention. Interestingly, the levels of DNA methylation at the *HOXA10* gene promoter were dramatically reduced in the PCOS group with the intervention (Figure 4). These findings are consistent with the higher expression of the *HOXA10* gene detected in the endometrium of PCOS + MET women compared to the PCOS group without intervention (Figure 3A).

## 3. Discussion

PCOS is one of the leading endocrine and metabolic disorders in premenopausal women, characterized by an alteration of the hypothalamic–pituitary–ovarian (HPO) axis, hyperandrogenism, anovulation, infertility, and insulin resistance, among other morbidities [7]. To date, the PCOS etiology remains unclear. It has been established that the endometrium is a reproductive tissue affected by PCOS partly by alterations in gene expression [39] that in turn are associated with epigenetic changes. Lifestyle intervention and metformin therapy exert positive metabolic and endocrine effects on women with PCOS and tissues affected by the disease, such as the endometrium [40]. In this preliminary study, we found that after intervention with metformin and a carbohydrate-controlled diet, the expression of the *HOXA10* (related to endometrial function) and *GAB1* and *SLC2A4* genes (associated with insulin signaling) increased in the endometrium of eight women with PCOS. The expression of these genes decreased in the endometrium of PCOS patients compared to women without the disease. Remarkably, the increased expression of the *HOXA10* gene in the endometrium of women undergoing this intervention was associated with decreased DNA methylation at its promoter. Nevertheless, we do not rule out the influence of the patients’ clinical background on the observed endometrial epigenetic modifications [41,42,43,44].

### 3.1. Effect of the Intervention with Metformin and a Carbohydrate-Controlled Diet in Metabolic and Clinical Variables in PCOS Women

According to the diagnostic criteria for the patients included in the present study, we confirmed that several clinical parameters were altered in women with PCOS compared to women from the control group, such as androstenedione, estradiol, and testosterone serum levels, as well as the LH/FSH ratio, FAI, and IR-HOMA index (Table 1). In contrast to previous reports, we found no statistically significant changes in some clinical parameters such as BMI, DHEA, progesterone, prolactin, SHBG, glucose, and insulin between PCOS women and controls [1]. The differences between the results obtained in the present study and previous reports could be due to the characteristic phenotypic heterogeneity of the disease, and the small sample size analyzed in the present study. In addition, we did not divide the PCOS group according to BMI, which influences the phenotype of these patients [45,46,47]. Although we did not observe a dramatic reduction in the clinical parameters of the PCOS group subjected to the intervention with metformin and a carbohydrate-controlled diet, we found that BMI, androstenedione, estradiol, insulin, and IR-HOMA index decreased after the intervention (Table 2). Our results confirm the positive effects of metformin and medical nutrition therapy (MNT) in the clinical parameters of women with PCOS, as previously reported [48]. Of note, the main limitation of this study is that only eight patients were subjected to the intervention. Further studies are necessary to confirm these preliminary findings in larger sample sizes and specific PCOS phenotypes. Another limitation of this study is that we did not separately compare the effect of metformin and the carbohydrate-controlled diet. It has been demonstrated that a dietary intervention or metformin therapy alone is sufficient to improve metabolic and endocrine parameters in women with PCOS [49,50,51]. Therefore, future studies should evaluate the effect of both interventions separately on the clinical and molecular characteristics of women with PCOS.

### 3.2. Effect of the Intervention with Metformin and a Carbohydrate-Controlled Diet in the Expression of Genes Related to Endometrial Function in the Endometrium of PCOS Women

The gene expression analyses in the present study showed that *HOXA10* gene expression decreased in the endometrium of PCOS women compared to controls (Figure 1A), as previously reported [14,15]. Interestingly, we observed an increase in the expression of the *HOXA10* gene in the patients subjected to metformin and dietary intervention (Figure 3A). These results highlight the potential benefit of the intervention used in the present study on PCOS patients, since the *HOXA10* gene is fundamental for the differentiation and receptivity of the endometrium [39]. The future assessment of gene expression (and DNA methylation) of endometrial receptivity markers such as *CDH6* (cadherin 6), L-selectin ligands, osteopontin and its receptor αvβ3 integrin, and *LIF* is necessary to provide more information about the association among PCOS pathogenesis, endometrium receptivity, and the metformin and carbohydrate-controlled diet used in the present study [52,53,54,55].

In contrast to previous studies, we observed no changes in the expression of the *ESR1* gene in the endometrium of women with PCOS compared to the endometrium of controls (Figure 1C) [21,56,57]. Unexpectedly, the metformin and dietary intervention induced the expression of *ESR1* in the endometrium of PCOS women (Figure 3B). This finding should be explored in future studies, as deregulation of *ESR1* expression is associated with endometrial hyperplasia and endometriosis [58,59,60,61].

We found no expression changes of *PAX6*, *ESR2*, *IGFBP1*, and *IRS1* genes among the study groups, probably due to the limitations of the present study since these genes have been reported to be differentially expressed in the endometrium of women with PCOS compared to women without the disease [9,22,23,62,63]. We also cannot discard the effect of medroxyprogesterone acetate on our findings, as it has been previously demonstrated that this synthetic hormone modifies gene expression in endometrial cells [64].

The results of the present study showed that *GAB1* and *SLC2A4* expression is decreased in the endometrium of women with PCOS compared to controls (Figure 1F,G), as previously reported [18,19]. Remarkably, the expression of both genes was induced after the metformin and dietary intervention (Figure 4C,D). In line with our results, previous studies have shown that *SLC2A4* expression is restored by metformin therapy in the endometrium of women and animal models of PCOS [34,65,66]. To the best of our knowledge, this is the first report about the effects of metformin and a carbohydrate-controlled diet on the expression of genes related to balanced energy homeostasis, which is fundamental for the correct functioning of the endometrium [39].

Our preliminary results suggest that the intervention used in the present study may have beneficial effects on the endometrium function of PCOS women, which should be explored in future functional studies.

### 3.3. Effect of the Intervention with Metformin and a Carbohydrate-Controlled Diet on the DNA Methylation Levels of Genes Related to Endometrial Function in the Endometrium of PCOS Women

Previous studies have reported alterations in the content of DNA methylation in tissues affected by PCOS [24,32]. To our knowledge, no studies have reported differential DNA methylation levels in the endometrium of women with PCOS and women without the disease [31,67]. To explore DNA methylation as a possible mechanism of gene expression regulation in response to the intervention used in the present study, we analyzed the enrichment of 5mC at the promoters of the genes of interest using MeDIP-qPCR.

*LINE-1* hypomethylation has been reported in peripheral blood cells and ovarian tissue of PCOS women. In contrast, hypermethylation of this genomic element has been found in the cumulus cells of these women [68,69,70]. In the present study, we did not detect changes in the levels of DNA methylation in the repetitive region *LINE-1*, neither in the endometrium of controls and PCOS women nor after the intervention. Moreover, we did not find differences in the enrichment of this epigenetic mark in the imprinted gene *H19* between the study groups (Figure 2A,B). Our findings suggest that global DNA methylation is not altered in the endometrium of PCOS women; however, this finding should be confirmed using other techniques, such as DNA methylation sequencing.

The *HOXA10* gene is hypermethylated in the endometrium of women with endometriosis and animal models of this disease, as well as in other endometrial diseases such as uterine polyps, intramural myoma, and submucosal myoma in which the DNA methylation status is negatively correlated with gene expression levels [71,72,73,74,75,76,77]. These reproductive diseases and PCOS are associated with infertility partly by an endometrial dysfunction that, in turn, may be explained by the increased DNA methylation at the *HOXA10* promoter. In the present study, we observed a significant increase in the enrichment of 5mC at the promoter of the *HOXA10* gene in the endometrium of women with PCOS compared to controls (Figure 2C). This finding was associated with a decrease in the expression of *HOXA10* in the endometrium of PCOS women (Figure 1A), which has been previously reported [14,15].

Lifestyle intervention is a promising alternative for managing PCOS by modulating the DNA methylation content in tissues affected by the disease [78,79]. Metformin is one of the main pharmacological lines of treatment against PCOS. It has been recently reported that metformin compensates for the effect of dehydroepiandrosterone on the DNA methylation levels of oocytes in an experimental animal model of PCOS [80]. In the present study, we have shown for the first time that metformin and a carbohydrate-controlled diet decreased the DNA methylation levels in the *HOXA10* gene promoter in the endometrium of PCOS women (Figure 4), which in turn was associated with an increase in its expression (Figure 3A). These preliminary findings suggest that in PCOS women, the beneficial effects of metformin and MNT in the endometrial expression of the *HOXA10* gene are partly mediated by DNA methylation. However, future studies should explore the mechanism involved, as it has been reported that metformin regulates DNA methylation by direct and indirect actions [37].

In contrast, we found no changes in the levels of *PAX6* DNA methylation in the endometrium of PCOS women subjected to the intervention. Moreover, we did not observe changes in the content of DNA methylation in the promoter of the *ESR1* gene that could explain the gene expression changes observed in our results. Further studies are required to explore the involvement of promoter mutations and other epigenetic mechanisms, such as histone post-translational modifications, in the gene expression changes found in the present study.

## 4. Materials and Methods

### 4.1. Inclusion and Exclusion Criteria for Patient Recruitment

This study was authorized and performed at the Instituto Nacional de Perinatología (INPer) in Mexico City (registry number 3000-20209-04-16). The recruitment of patients took place between 2018 and 2019. Fifteen patients diagnosed with PCOS and eight healthy women (control group) were recruited. For both groups, inclusion criteria were as follows: 18 to 45 years old (reproductive aged-women), BMI < 35 kg/m^2^, without prescription of hormonal treatment at least two months before participating in the study, and signing a written form of informed consent. Based on the Rotterdam Criteria, the inclusion criteria for PCOS patients comprised infertility, hyperandrogenism, oligo or anovulation, and polycystic ovary phenotype [5]. The inclusion criteria for women in the control group were regular menstrual cycles without PCOS symptoms. From the control and PCOS group, subjects with hormonal or GnRHa treatments, cancer diagnosis, radiotherapy or chemotherapy, chronic hypertension, diabetes mellitus, cardiac, hepatic, or renal diseases, hypo or hyperthyroidism, and asthma were excluded. Patients with infertility not related to PCOS were excluded from the PCOS group.

### 4.2. Sample Collection and Metabolic Intervention

Blood samples were obtained via vein puncture during the early proliferative phase of the menstrual cycle and stored in vacuum blood collection tubes with serum-separating gel; serum was further isolated via centrifugation at 1300× *g* at 4 °C for 10 min and stored at −70 °C. Endometrial tissue was collected using a Pipelle suction curette during the mid-proliferative phase of the menstrual cycle and stored in RNAprotect Tissue Reagent (cat. no. 76106, Qiagen, Valencia, CA, USA) at −20 °C. Before sample collection, all PCOS patients received 10 mg/day of medroxyprogesterone acetate for ten days (Provera, Pfizer, New York, NY, USA) to induce menstruation. This study was divided into two phases. In the first part of the study, we compared clinical characteristics, gene expression, and DNA methylation data between 15 women diagnosed with PCOS and 8 controls. Then, eight of the PCOS patients underwent a three-month pharmacologic intervention with oral metformin 1500 mg/day (Dabex XR, Merck, Darmstadt, Germany) and MNT with a clinical nutritionist to avoid significant variations in food intake among them (PCOS + MET group). A nutrition assessment was performed, and energy requirements were estimated with the Mifflin-St Jeor et al. equation [81] using current body weight. A controlled carbohydrate-restricted diet (40–45% of total energy intake), with 20–25% of energy from proteins and 30–35% from fat, was prescribed. The recommended diet focused on high-quality carbohydrate foods (high fiber and/or low glycemic index) and promoted the intake of fruits, vegetables, low-fat dairy, legumes, whole grains, oily fish and food sources of monounsaturated fatty acids (avocados, canola oil, seeds), while limiting the intake of added sugars and ultraprocessed foods. Education themes included the healthy eating plate, healthy carbohydrates, basic carbohydrate counting, portion size estimation, and improving food choices to reduce ultra-processed foods (usually high in added sugars and/or high glycemic index). Samples from PCOS patients were obtained before and after three months of pharmacologic and dietary intervention.

### 4.3. Measurement of Hormone and Glucose Serum Levels

Automated immunoassays (Immulite 2000 system, Siemens Healthcare Diagnostic, Erlangen, Germany) were performed to determine serum levels of androstenedione (cat. no. L2KAO2), dehydroepiandrosterone (DHEA, cat. no. L2KDS2), estradiol (cat. no. L2KE22), follicle-stimulating hormone (FSH, cat. no. L2KFS2), insulin (cat. no. L2KIN2), luteinizing hormone (LH, cat. no. L2KLH2), prolactin (cat. no. L2KPR2), progesterone (cat. no. L2KPW2), testosterone (cat. no. L2KTW2), and SHGB (cat. no. L2KSH2) as specified by the manufacturer. Fasting glucose levels were determined using a Dri-Chem Slide Glu-P III kit (cat no. 15809528, Fujifilm, Tokyo, Japan). The free androgen index (FAI) was calculated by dividing the total testosterone level by the SHBG level and multiplying by 100 [82]. The insulin resistance index was determined with the Homeostatic Model Assessment of Insulin Resistance (HOMA-IR) using the formula: fasting glucose level (mg/dL) × fasting insulin level (µIU/mL)/405 [83].

### 4.4. DNA and RNA Isolation

Genomic DNA and total RNA were isolated with an AllPrep DNA/RNA Mini kit (cat. no. 80204, Qiagen, Valencia, CA, USA), following manufacturer instructions. DNA and RNA integrity was analyzed using 1% agarose gel electrophoresis.

### 4.5. RT-qPCR

cDNA synthesis was carried out using the SuperScript III First-Strand Synthesis SuperMix (cat. no. 18080400, Invitrogen, Carlsbad, CA, USA), as specified by the supplier. cDNA was subjected to PCR to amplify a gene fragment of *HOXA10*, *PAX6*, *ESR1*, *ESR2*, *IGFBP1*, *GAB1*, *SLC2A4*, and *IRS1*. *ACTB* (actin beta) was used as an internal control of constitutive expression. The sequences of the specific primers are listed in Table S1 of Supplementary Materials. SYBR Green Master Mix (cat. no. 4309155, Applied Biosystems, Foster City, CA, USA) was used as the detection method in a StepOne Plus Real-Time PCR system (cat. no. 4376357, Applied Biosystems, Foster City, CA, USA) following cycling conditions specified by the manufacturer. Relative quantification was performed with the ΔΔCt method [84].

### 4.6. Methylated DNA Immunoprecipitation (MeDIP)-qPCR

MeDIP was carried out as previously described [85], with minor modifications. Shearing of 2 µg of DNA was performed in a Bioruptor Pico sonicator (cat. no. B01060010, Diagenode, Seraing, Belgium) by 30 cycles of 30 s ON and 30 s OFF at 20–60 kHz to obtain DNA fragments with a modal size of 200 bp, which was confirmed on a 1.5% *w*/*v* agarose gel stained with GelRed (cat. no. 41003, Biotium, Fremont, CA, USA). 1 µg of sonicated DNA was diluted in TE buffer (10 mM Tris, 0.1 mM EDTA, pH 8.0), denatured for 10 min at 100 °C, and snap-chilling on wet ice. 10% *v*/*v* of the sample was transferred to a clean tube for the input (normalization control). Diluted DNA was incubated with immunoprecipitation buffer (IP, 10 mM Na_2_HPO_4_, 10 mM NaH_2_PO_4_, 140 mM NaCl, 0.05% *v*/*v* Triton X-100) and 1 μg of anti-5-methylcytosine (5mC) antibody (cat. no. C15200081, Diagenode, Seraing, Belgium) at 4 °C overnight on a rotating wheel. Non-specific mouse IgG was used as a negative control antibody (cat. no. C15400001, Diagenode, Seraing, Belgium). The antibody-DNA complexes were incubated with BSA-blocked magnetic beads (Dynabeads Protein G, cat. no. 10003D Invitrogen, Carlsbad, CA, USA) in rotating agitation at 4 °C for 1 h and washed with IP buffer. Samples were incubated with digestion buffer (50 mM Tris pH 8.0, 10 mM EDTA, 0.5% *w*/*v* SDS) and Proteinase K (cat. no. 25530049, Invitrogen, Carlsbad, CA, USA) overnight at 55 °C in agitation (300 rpm) to release the DNA from the beads. DNA purification was performed using phenol-chloroform-isoamyl alcohol 25:24:1 (cat. no. P2069, Sigma-Aldrich, Merck, Darmstadt, Germany) and diluted in TE buffer. qPCR with the immunoprecipitated DNA was performed using the SYBR Green method in the StepOne Plus Real-Time PCR system. In every MeDIP experiment, *H19* and *LINE-1* were used as endogenous methylated controls, and *GAPDH* promoter was used as an endogenous non-methylated control. Primers used are enlisted in Table S1. Relative levels of 5mC enrichment were determined using the ΔΔCt method and normalized to IgG control.

### 4.7. Statistical Analysis

Statistical analysis was performed using GraphPad Prism 8.4.3 software (Graph Pad Software, San Diego, CA, USA). Experimental data are presented as the mean with standard deviation (SD) or standard error (SE) from three or more independent experiments. One-way ANOVA tests were performed, followed by the Tukey post hoc test. Paired data (PCOS patients before and after dietary and pharmacological intervention) were analyzed with the paired Student *t*-test. Kruskal-Wallis, Dunn’s, and Wilcoxon signed rank tests were performed for data without normal distribution. Statistical differences were considered when *p* ≤ 0.05.

## 5. Conclusions

In the present study, we have shown that gene expression of *HOXA10*, *GAB1*, and *SLC2A4* decreased in the endometrium of PCOS patients compared to women without the disease. Remarkably, the expression of these genes increased after metabolic intervention with metformin and a carbohydrate-controlled diet. Moreover, increased *HOXA10* expression after the intervention was associated with decreased DNA methylation levels at its promoter. Our preliminary findings suggest that this intervention improves endometrial function in PCOS women by modulating DNA methylation and gene expression of genes associated with endometrial receptivity and insulin signaling. Further investigation in larger sample sizes is warranted to elucidate the beneficial effects of metformin and dietary intervention on the endometrium of women with PCOS.

## Figures and Tables

**Table 1:** Demographic and clinical characteristics of healthy women (CONTROL) and PCOS patients who participated in this study.

Variable	CONTROL (*n* = 8)	PCOS (*n* = 15)
Age (years old)	27.80 ± 5.1	27.70 ± 4.5
BMI (kg/m^2^)	25.50 ± 3.2	27.11 ± 4.7
Androstenedione (ng/mL)	2.08 ± 0.60	3.57 ± 1.45 *
DHEA (µg/dL)	150.80 ± 79.92	161.00 ± 94.98
Estradiol (pg/mL)	39.30 ± 15.76	62.67 ± 12.75 *
FSH (mIU/mL)	5.59 ± 1.58	5.40 ± 1.25
LH (mIU/mL)	4.42 ± 2.11	7.01 ± 3.26
LH/FSH	0.80 ± 0.41	1.28 ± 0.40 *
Progesterone (ng/mL)	0.28 ± 0.07	0.25 ± 0.06
Prolactin (ng/mL)	11.53 ± 4.56	10.17 ± 3.53
SHBG (nmol/L)	44.8 ± 27.32	28.57 ± 12.34
Testosterone (nmol/mL)	0.81 ± 0.13	1.49 ± 0.40 *
FAI index	2.21 ± 0.93	6.04 ± 2.96 *
Glucose (mg/dL)	82.17 ± 8.19	89.54 ± 11.10
Insulin (µIU/mL)	5.17 ± 3.10	15.67 ± 11.37
HOMA-IR index	1.04 ± 0.56	3.51 ± 2.62 *

Data are shown as mean ± SD; * *p* ≤ 0.05 vs. CONTROL. Body mass index, BMI; dehydroepiandrosterone, DHEA; follicle-stimulating hormone, FSH; luteinizing hormone, LH; sex hormone-binding globulin, SHBG; free androgen index, FAI; Homeostatic Model Assessment of Insulin Resistance, HOMA-IR.

**Table 2:** Hormonal and metabolic variables improved in PCOS patients after metabolic intervention.

Variable	PCOS (*n* = 8)	PCOS + MET (*n* = 8)
BMI (kg/m^2^)	28.51 ± 4.68	27.08 ± 5.06 *
Androstenedione (ng/mL)	4.46 ± 1.60	3.41 ± 0.98 *
Estradiol (pg/mL)	69.73 ± 10.42	59.80 ± 10.16 *
Insulin (µIU/mL)	21.45 ± 11.07	11.32 ± 7.13 *
HOMA-IR index	4.90 ± 2.74	2.39 ± 1.43 *

Data are shown as mean ± SD; * *p* ≤ 0.05 vs. PCOS. Body mass index, BMI; Homeostatic Model Assessment of Insulin Resistance, HOMA-IR.

## Data Availability

The original contributions presented in the study are included in the article; further inquiries can be directed to the corresponding author.

## Supplementary Materials

The following supporting information can be downloaded at: https://www.mdpi.com/article/10.3390/ijms24076857/s1.
