# Supplementary material for: The Effect of Metformin and Carbohydrate-Controlled Diet on DNA Methylation and Gene Expression in the Endometrium of Women with Polycystic Ovary Syndrome
Source: Int J Mol Sci. 2023 Apr 6;24(7):6857. doi: 10.3390/ijms24076857 (PMC10094785; doi:10.3390/ijms24076857)
Supplement: Supplementary file 1 [file ijms-24-06857-s001.zip › ijms-2214445-supplementary.pdf]

Table S1. List of primers used in this study.

	Gene	Primer sequence (5'-3′)	Amplicon size (bp)
**Analysis**			
Relative expression	*HOXA10*	F: CTCGCCGGAGAAGGATTC	90
		R: TCACTTGTCTGTCCGTGAGG	
	IGFBP1	F: TCCTTTGGGACGCCATCAGTAC	133
		R: GATGTCTCCTGTGCCTTGGCTA	
	ESR1	F: CACCAACCAGTGCACCATTG	205
		R: AAGGTTGGCAGCTCTCATGTC	
	ESR2	F: CCATGATCCTGCTCAATTCC	127
		R: CTCTTGGCAATCACCCAAAC	
	PAX6	F: AACGATAACATACCAAGCGTGT	120
		R: GGTCTGCCCGTTCAACATC	
	IRS1	F: AGCATCAGTTTCCAGAAGCAG	84
		R:GCTGAGGTCATTTAGGTCTTCA	
	GAB1	F: GGTGGTGAAGTGGTCTGCTC	87
		R: GAACCATCTCCTCTTCCATGCA	
	SLC2A4	F: CGAGCAACTTCATCATTGGCAT	79
		R: ACCGCAAATAGAAGGAAGACGT	
	ACTB	F: CTTCCTGGGCATGGAGTC	89
		R:CGTACAGGTCTTTGCGGATG	
MeDIP (gene promoter)	*HOXA10*	F: CGTCTCCTGGCCCATCAATA	132
		R: CCAATCCCGAGCCAGAGTTT	
	IGFBP1	F: GCGCTGCCAATCATTAACCTC	169
		R: CAAGTGATGGTGGAGCGGTG	
	ESR1	F: CTCGGGCTGTGCTCTTTTTC	113
		R: GTGTGGAGGGTCATGGTCAT	
	ESR2	F: TCTGTGCGCCACTATCCTTG	141
		R: AGAGGGGAGTAGTGCCTGAG	
	PAX6	F: TATTTTGTGTGAGAGCGAGCGG	108
		R: CTGAACCAGAGCGGGAAATGA	

GAB1	F: GCTCGCGTTCTGTTCAGGTT	119
	R: AACCTAGCTCTCCTGGTCGC	
IRS1	F: CCTCCAGCCCTGTTTGCATG	147
	R: CACGTCCGAGAAGCCATCG	
SLC2A4	F: TCAGAGACTCCAGGATCGGTT	97
	R: CGGAGCCTATCTGTTGGAAGC	
*LINE-1**	F: TTCCCTTTCCGAGTCAAAGA	71
	R: CAATATTCGGGTGGGAGAGA	
*H19**	F: GAGCCGCACCAGATCTTCAG	134
	R: TGATCACAGTGTGTTCCACCAA	
GAPDH	F: CCTTCCCCTAGTCCCCAGAAAC	122
	R: GCTCCTCTGCGACACGT	

*Used as controls of global methylation (*LINE-1*, repeated sequence; *H19*, imprinted gene).
